# DenvInt: A database of protein–protein interactions between dengue virus and its hosts

**DOI:** 10.1371/journal.pntd.0005879

**Published:** 2017-10-19

**Authors:** Lopamudra Dey, Anirban Mukhopadhyay

**Affiliations:** 1 Department of Computer Science and Engineering, Heritage Institute of Technology, Kolkata, West Bengal, India; 2 Department of Computer Science and Engineering, University of Kalyani, Kalyani, West Bengal, India; George Mason University, UNITED STATES

## Introduction

Dengue fever and associated dengue hemorrhagic fever are emerging globally as the most important arboviral disease for human population [1]. The dengue viruses (DENV) are members of the genus *Flavivirus* in the family Flaviviridae. Flaviviruses are arthropod-borne viruses, or arboviruses, which means they need an insect as a host to complete their life cycle. The full life cycle of dengue fever virus evolves the role of mosquito as a transmitter and human as the main victim and source of infection [2]. When a mosquito bites a person who has DENV in his or her blood, the mosquito becomes infected with the DENV. An infected mosquito can later transmit that virus to healthy people by biting them.

This dengue disease is rapidly spreading in all regions of WHO in recent years. Recently, one dengue vaccine was licensed, Dengvaxia (CYD-TDV), developed by Sanofi Pasteur (http://www.who.int/immunization/research/development/dengue_q_and_a/en/). But it is only applicable for use in individuals 9–45 years of age, and the vaccine is currently not prequalified. Therefore, intensive efforts to develop a vaccine to protect against dengue are still ongoing. It is primarily transmitted by 1 mosquito species, *Aedes aegypti*. The virus is transmitted to humans through the bites of infected female mosquitoes. After virus incubation for 4–10 days, an infected mosquito is able to transmit the virus for the rest of its life.

**Fig 1:**
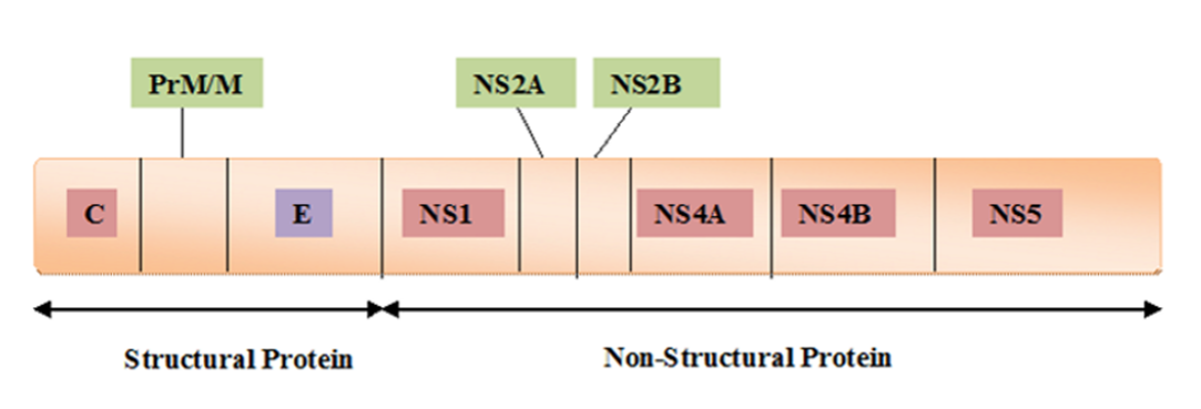
Shows dengue virus proteins.

In 1943, Ren Kimura and Susumu Hotta first quarantined the DENV [3]. These 2 scientists were studying blood samples of patients taken during the 1943 dengue epidemic in Nagasaki, Japan. One year later, Albert B. Sabin and Walter Schlesinger independently isolated the DENV [4]. The DENV genome is a single strand of RNA. It is referred to as positive-sense RNA because it can be directly translated into proteins. The viral genome encodes 10 proteins, as shown in Fig 1. Three are structural proteins, which are the capsid (C), the precursor of membrane protein (PrM/M), and the envelope protein (E), while the rest are nonstructural (NS) proteins:NS1, NS2A, NS2B, NS3, NS4A, NS4B, and NS5. These NS proteins play roles in viral replication and assembly.

Because the dengue genome encodes only 10 viral proteins, the virus needs to hijack host proteins to help its replication. An understanding of how viruses interact with host cellular machineries to survive and replicate is important for the development of drugs. One way that viruses interact with their hosts is by protein–protein interactions (PPI). Therefore, indentifying PPI between DENV and host proteins will increase understanding of the function of virus proteins and how they reproduce and cause disease. Several studies have focused on dengue–host protein interactions. In one study [5], Mairiang et al. identified 102 dengue–mosquito interactions and 46 dengue–human interactions by using yeast 2-hybrid screens. In another study [6], Khadka et al. used yeast 2-hybrid screens to discover 139 protein interactions that involve 105 human proteins and 8 dengue serotype 2 proteins. Folly et al. [7] applied a bacterial 2-hybrid screen test to detect 31 dengue-interacting human proteins. In yet another study [8], Doolittle et al. predicted 4,376 dengue–human and 176 dengue–mosquito protein interactions based on structural similarity. Le Breton et al. [9] used yeast 2-hybrid screening to identify PPI between 108 human proteins and the NS3 and NS5 proteins of dengue serotype 2. In another study [10], Tham et al. identified 42 dengue–mosquito interactions by using yeast 2-hybrid test. Dengue infections are caused by 4 closely related viruses named DENV-1, DENV-2, DENV-3, and DENV-4, which are found in Asia, Africa, and North America [11]. These 4 viruses are called serotypes because each has different interactions with the antibodies in human blood serum. Some dengue–host interactions are serotype specific. In another study [12], Balmaseda et al. conducted a cross-sectional study on hospitalized children with confirmed DENV infections that shows that DENV-2 and DENV-3 serotypes are more life-threatening than the other serotypes, and DENV-4 is responsible for a milder illness.

Currently, there exist a few DENV–related databases like DENVDB (http://proline.bic.nus.edu.sg/denvdb) and DEnvirDB (http://www.ladydoakcollege.edu.in/denvirdb/index.php), which store different information about DENV. DENVDB is developed by the National University of Singapore, which provides the protein sequences of DENV. In addition, the DENVDB provides information on the protein alignment, fully conserved sequences within individual dengue proteins, BLAST features, and variant analysis. DENVirDB has been developed to provide sequence information along with the computational annotation [11]. There are some other databases, such as Flavitrack and DengueInfo, that consist of genome sequences, along with sequence analysis tools, such as pairwise and multiple sequence alignment [11]. However, VirusMentha (http://virusmentha.uniroma2.it/) and VirHostNet (http://virhostnet.prabi.fr/) provide virus–virus and virus–host interaction networks but not exclusively concentrated on specific organisms [13][14]. Both of the databases offered little information on dengue–human and dengue–mosquito PPI. The VirHostNet database describes significant virus interactions, but the data set has not been periodically updated since its publication [13]. Therefore, no dengue host–pathogen PPI database has been available until now, to the best of our knowledge. Motivated by this, here the DenvInt database is introduced, which provides both dengue–human and dengue–mosquito protein interactions because DENV requires both human and mosquito to complete its life cycle. The database provides annotation type, National Center for Biotechnology Information (NCBI) gene ID of the host proteins, experimental methods, and PubMed publication ID (PMID) of each interaction. Furthermore, all host protein interactions with all 4 dengue serotypes are either serotype specific or serotype independent. Therefore, dengue serotype information for each dengue–host interaction is mentioned in the database. All the interactions contained in DenvInt are manually curated from published papers. We have considered only those protein interactions that are identified by high-throughput screens and are biologically relevant. The database will be regularly updated every month to keep it uptodate. DenvInt is freely accessible at https://denvint.000webhostapp.com, and the complete database is available for download.

## Materials and methods

DenvInt integrates contextual information concerning interacting proteins between dengue and its hosts—both human and mosquito. All the interactions are manually curated from published peer-reviewed journals and virus databases such as VirHostNet and VirusMentha. It stores only those PPI that pass through a very strict filtering procedure to maintain a high-quality PPI repository. We have considered different experimental methods like yeast 2-hybrid test (Mairiang et al. [15], [7], [9], Khadka et al. [6]), bacterial 2-hybrid test (Folly et al. [7]), complex pull down assay and coaffinity purification (co-AP)assay (Mairiang et al. [5]), colocalization (Balinsky et al. [16]), and in vitro pull down assay (Chiu et al. [17]) to detect the PPI. No computationally predicted interaction is mentioned in the database. Most of the DENV interactions are serotype specific. During curation, dengue serotypes are investigated for each interaction along with experimental methods. Therefore, all the interactions in DenvInt are annotated with associated interaction type, dengue serotype, experimental procedure, paper name, author name, and PMID. The dengue–human and dengue–mosquito PPI are given in S1 File and S2 File, respectively. NCBI gene symbols of human genes and mosquito genes are chosen to cross-reference all host proteins, which provides protein ID, gene name, and gene symbol or alias defined in different genome reference databases (ENSEMBL, UNIPROT, NCBI, INTACT, HPRD, etc.). This cross-referencing of proteins will help to prepare nonredundant protein–protein interactions defined in different databases. A user-friendly, interactive public web repository based on MySQL and Hypertext Preprocessor (PHP) is developed to publish the dengue–host interactions. Users can query the database by choosing a dengue protein from a list of 10 dengue proteins(C, E, PrM/M, NS1, NS2A, NS2B, NS3, NS4A, NS4B, NS5) or a host protein from a list of human and mosquito proteins, and the query will return a dengue–host PPI list with dengue serotype, host protein, NCBI gene ID of the protein, and PMID. Another important feature of this web interface is the updating procedure. Newly discovered interactions can be easily updated on the website by using asimple Structured Query Language (SQL) query update.

### Database specification

**Fig 2:**
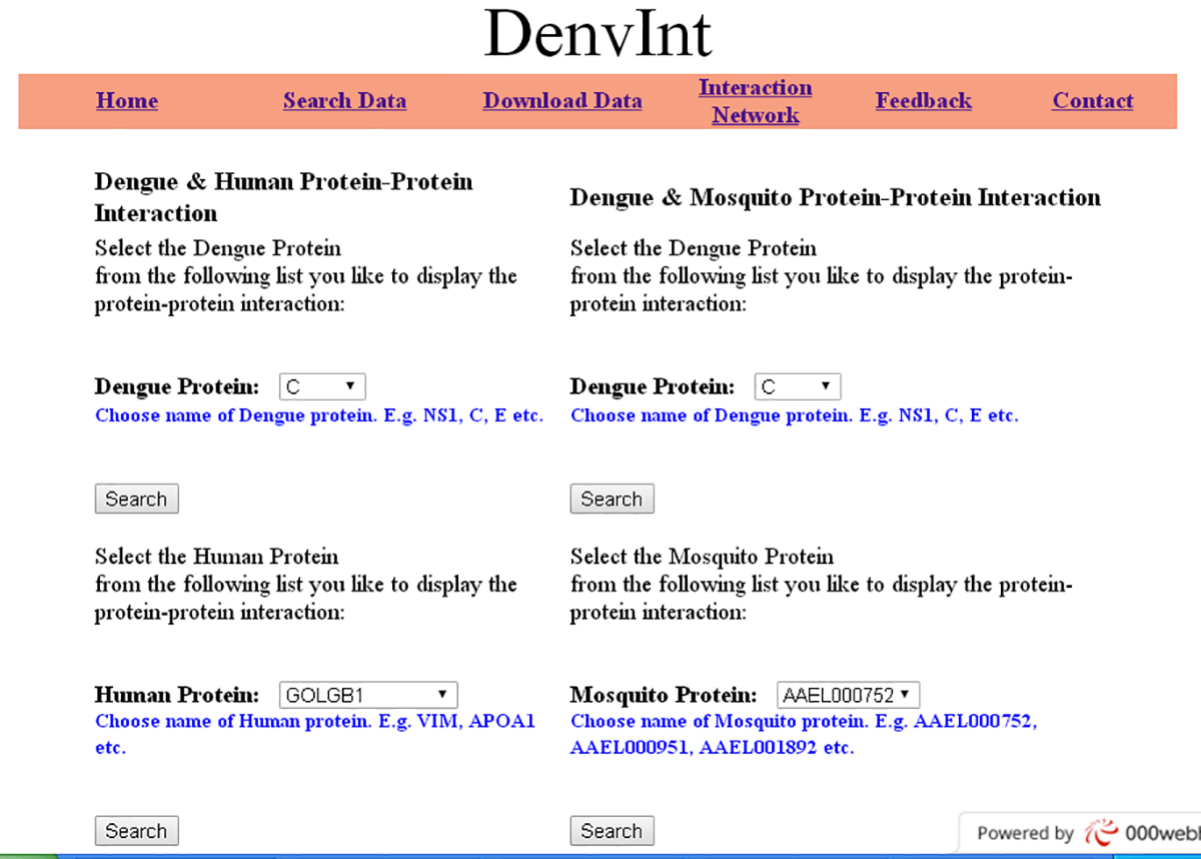
Screenshot of user query interface of DenvInt database. User can retrieve interaction data by selecting a dengue protein or a host protein from the list.

**Fig 3:**
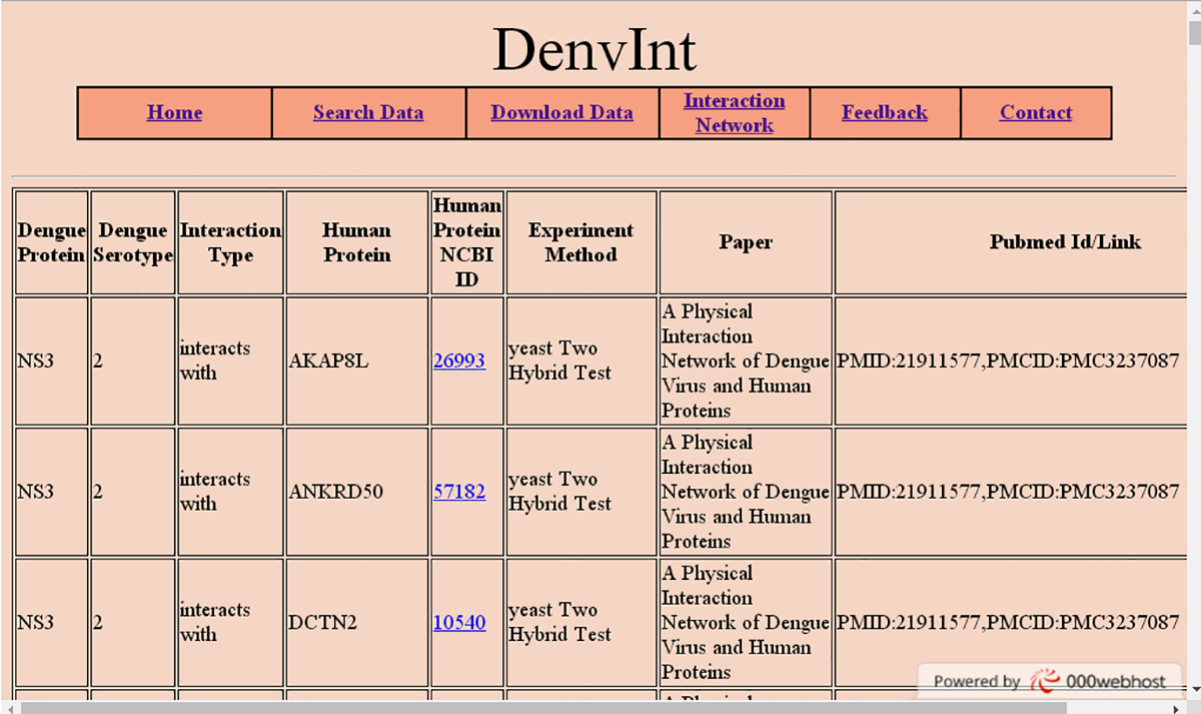
Screenshot of user query interface of DenvInt database. User chooses dengue protein NS3 and all PPI with human proteins; and dengue serotype, interaction type, NCBI ID, paper title, and PMID are retrieved and presented in the web page. NCBI ID, National Center for Biotechnology Information ID; PMID, PubMed ID; PPI, protein–protein interactions.

DenvInt is a web-based biological repository that provides comprehensive information on PPI between dengue–human and dengue–mosquito. MySQL and PHP are used to design a dynamic web interface and create a relational database to store information. To design the front-end graphical user interface of our website, we have used HTML with CSS and PHP technologies. The database is freely available to users. Two tables are prepared in the database—one for dengue–human and another for dengue–mosquito protein interactions. Each table contains fields such as dengue protein, dengue serotype, interaction type, host protein, NCBI gene ID, experiment method, paper title, author name, and PMID. The "search data" menu contains 4 search buttons corresponding to dengue–human PPI and dengue–mosquito PPI (Fig 2). Two search buttons help users to choose a particular dengue protein from a list of 10 dengue proteins and retrieve interactions as well as contextual data associated with it. Another 2 search buttons are used to select a host protein (human protein or mosquito protein) from a list of host proteins that turn up a host–dengue PPI. The NCBI gene IDs of both human and mosquito proteins are hyperlinked with the website http://www.ncbi.nlm.nih.gov. Therefore, users can also view gene-related information, such as official symbol, official full name, lineage, aliases, etc., by clicking on the NCBI gene ID attribute of both human and mosquito proteins (Fig 3). However, users can download the total interaction list from the "download" menu. The "feedback" menu on the website contains a form to collect suggestions from the researchers on new PPI or on existing interactions. After we verify the possible interaction, they can be immediately added in the database.

### Database utility

The database integrates all possible DENV–related PPI with both hosts (human and mosquito). It provides facility to the researchers to know which human proteins are affected when DENV enters the human body. This information can lead to antiviral drug discovery by analyzing infected protein pathways. The database is enriched with the dengue serotype, annotation type, and experiment method of each interaction. The information would be helpful in understanding the mechanism of the viral pathogenesis process. Researchers can also apply computational methods to predict PPI by using presently available data, which may be further experimentally investigated for validation.

### Summary of the PPI

Dengue–human PPI is composed of 535 nonredundant interactions between 335 different human proteins and 10 dengue proteins. Dengue–mosquito PPI is composed of 249 nonredundant interactions between 140 different mosquito proteins and 10 dengue proteins [18, 19]. In this section, we give a summary of the interactions with respect to different metrics from a network-analysis point of view. We have used the Cytoscape statistical environment to compute network connected components, density, diameter, and shortest path measures of the network [20].

Here, both networks are modeled as bipartite graphs, in which nodes represent the proteins, and edges represent the interactions. The edges of the PPI networks are treated as undirected. Before analyzing the networks, some network parameters should be known, and they are explained below.

1. Network connected component: The number of connected components indicates the connectivity of a network—a lower number of connected components suggests a stronger connectivity.
2. Network diameter: The network diameter is the maximum path length between 2 nodes in the network.
3. Network density: The network density shows how densely the network is populated with edges, ignoring self-loops and duplicated edges. The density ranges from a value between 0 and 1. A network that contains no edges and solely isolated nodes has a density of 0. In contrast, the density of a clique is 1 [21].
4. Network centralization: Network centralization of a network is close to 1 if it looks like a star, and for a decentralized network, the value is close to 0.
5. Network heterogeneity: The network heterogeneity parameter shows the tendency of a network to incorporate hub nodes. Study shows that biological networks tend to be heterogeneous. Few hub nodes are extremely connected, while the majority of nodes have very few connections [22].
6. Average number of neighbors: The average number of neighbors indicates the average connectivity of a node in the network. It denotes the average number of nodes connected with each host node.

Table 1 indicates the values of all parametes of the dengue–human PPI network and dengue–mosquito PPI network [23], and it is found from the table that the number of interactions in the first case is higher. It is evident from the table that the dengue–mosquito PPI network has a higher density and average number of neighbors compared with the dengue–human PPI network, but the dengue–human PPI network is more heterogeneous, i.e., it contains more subnodes than the dengue–mosquito PPI network. These are certainly not the expected values; these are simply an indication of how the networks are structured, which may be useful information to the users for further analysis of the networks.

**Table 1:** Comparative statistical analysis of dengue–human and dengue–mosquito PPI network.

Parametes	Dengue–Human Network	Dengue–Mosquito Network
Network connected component	1	1
Network diameter	6	8
Network density	0.008	0.022
Network centralization	0.460	0.443
Network heterogeneity	3.934	2.534
Average number of neighbors	2.917	3.302

Abbreviation: PPI, protein–protein interactions.

## Results and discussion

**Fig 4:**
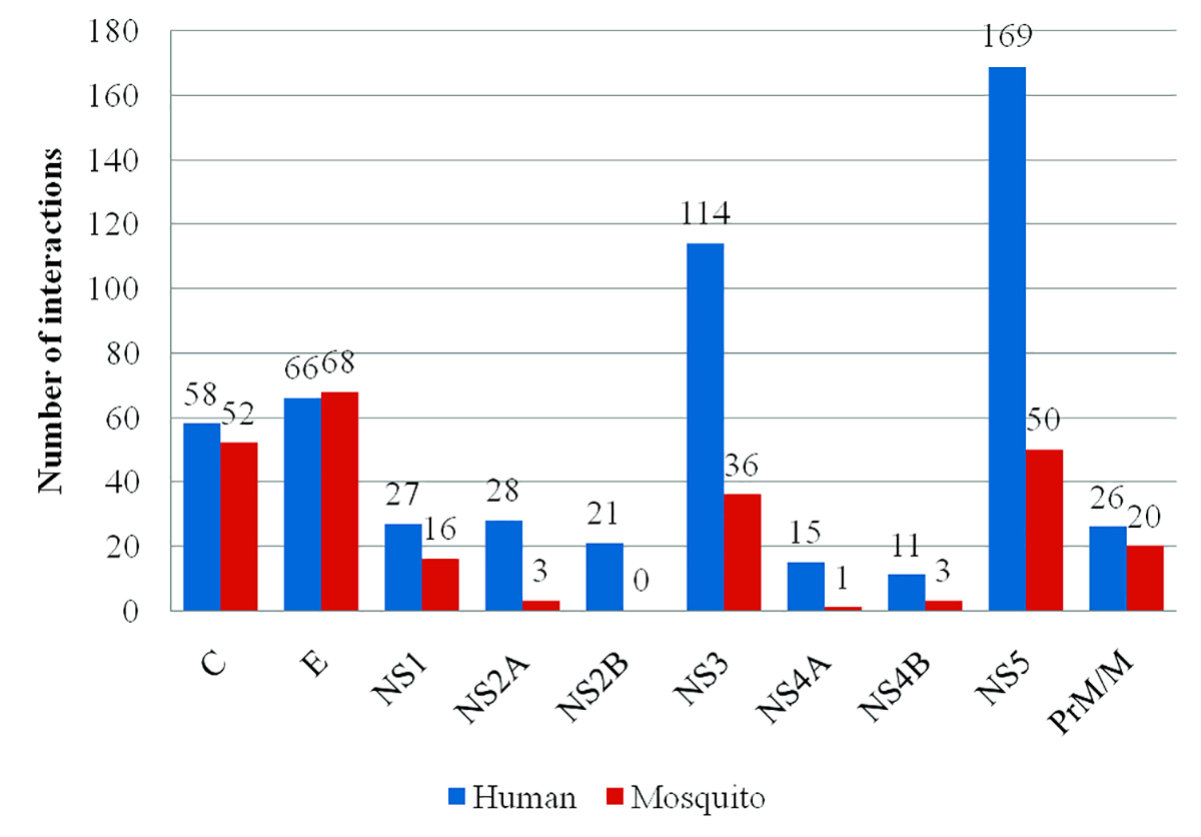
Dengue virus proteins’ frequency of interaction with human proteins and mosquito proteins. The dengue protein NS5 interacts with maximum number of human proteins (169), and dengue protein E interacts with maximum number of mosquito proteins (68).

**Fig 5:**
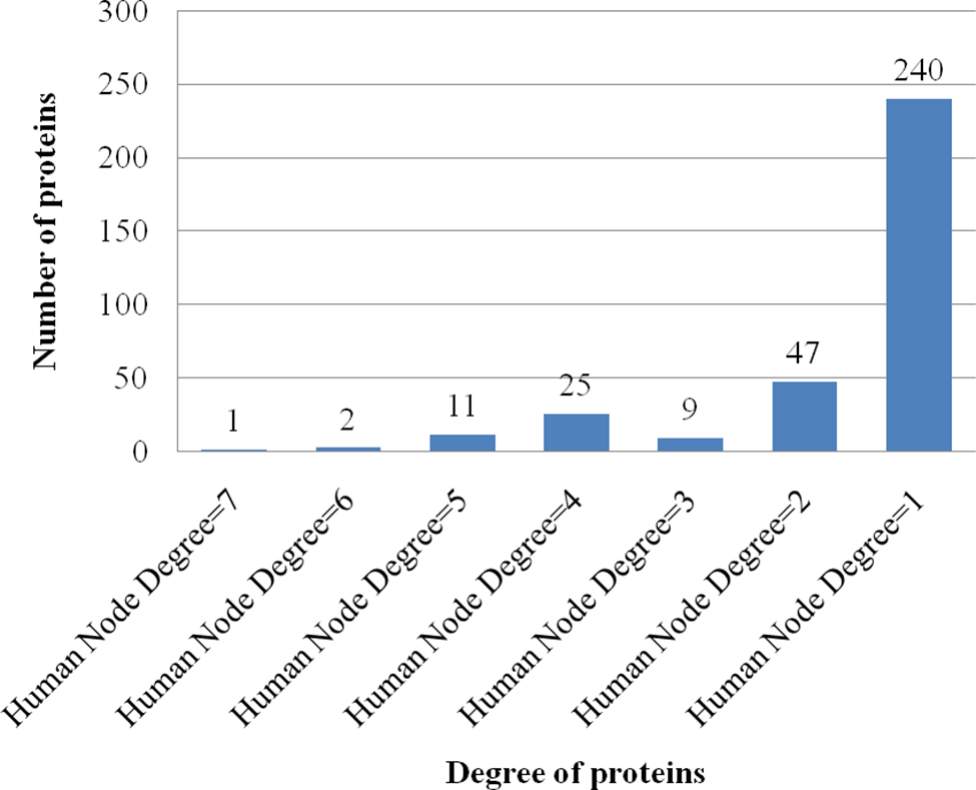
Average degree distribution of human protein nodes in dengue–human PPI network. It can be noted from the figure that at human node degree = 1, there is a maximum number (240) of interactions, and at human node degree = 7, there is only 1 interaction. Therefore, most of the human proteins interact with 1 dengue protein, and a majority of human proteins participate in a few interactions. PPI, protein–protein interactions.

**Fig 6:**
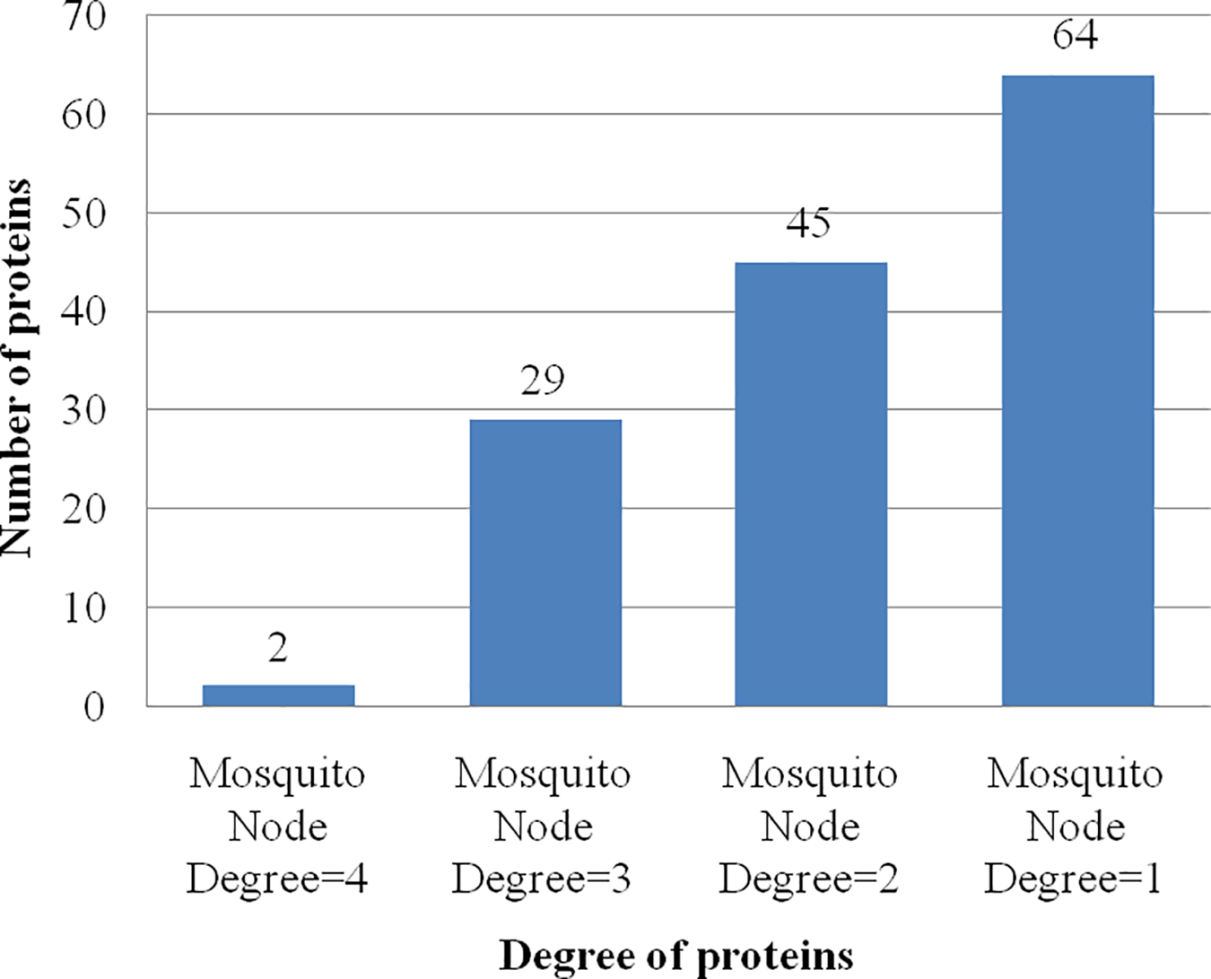
Average degree distribution of mosquito protein nodes in dengue–mosquito PPI network. It can be noted from the figure that at mosquito node degree = 1, there is a maximum number (64) of interactions, and at human node degree = 4, there are only 2 interactions. Therefore, most of the mosquito proteins interact with 1 dengue protein, and a majority of mosquito proteins participate in a few interactions. PPI, protein–protein interactions.

The DenvInt database consists of 784 unique interactions, including 535 dengue–human PPI and 249 dengue–mosquito PPI. There are 335 different human proteins and 140 different mosquito proteins present in the dengue–human and dengue–mosquito PPI database, respectively. These interactions involved 10 viral bait proteins, including C, E, PrM/M, NS1, NS2A, NS2B, NS3, NS4A, NS4B, and NS5. The first observation of the PPI network is protein degree, which defines the number of interacting partners of a protein. The number of host interactions for each dengue protein identified in this manually curated database is shown in Fig 4. It is evident from the figure that dengue protein NS5 and E interact with the maximum number of human proteins (169) and the maximum number of mosquito proteins (68), respectively. No interaction is identified between dengue protein NS2B and mosquito protein. The average node degree distribution expresses the number of nodes with degree d for *d* = 0, 1,…, *n*. Each node represents a single interaction between a dengue protein and a host protein. Therefore, if a host protein interacted with 3 different dengue proteins, this would have a degree equal to 3. The average degree distribution of human protein nodes in the dengue–human PPI network is shown in Fig 5, and the average degree distribution of mosquito protein nodes in the dengue–mosquito PPI network is shown in Fig 6. Most of the PPI networks are characterized by scale-free property of the degree distribution, for which a few proteins interact with a large number of proteins, and a majority of proteins participate in a few interactions [24]. Here, from Fig 5, it is noticeable that among the 335 human proteins, only 1 human protein, HBA1, has degree 7 because it interacts with 7 dengue proteins, whereas 240 human proteins interact with 1 dengue protein. The list of degree of 335 human proteins is given in S3 File. A similar pattern is also visible in Fig 6, in which only 2 mosquito proteins have degree 4, and 64 mosquito proteins have degree 1. The list of degree of 140 mosquito proteins is given in S4 File. Therefore, both dengue–host PPI databases support the scale-free property of the protein interaction network.

**Fig 7:**
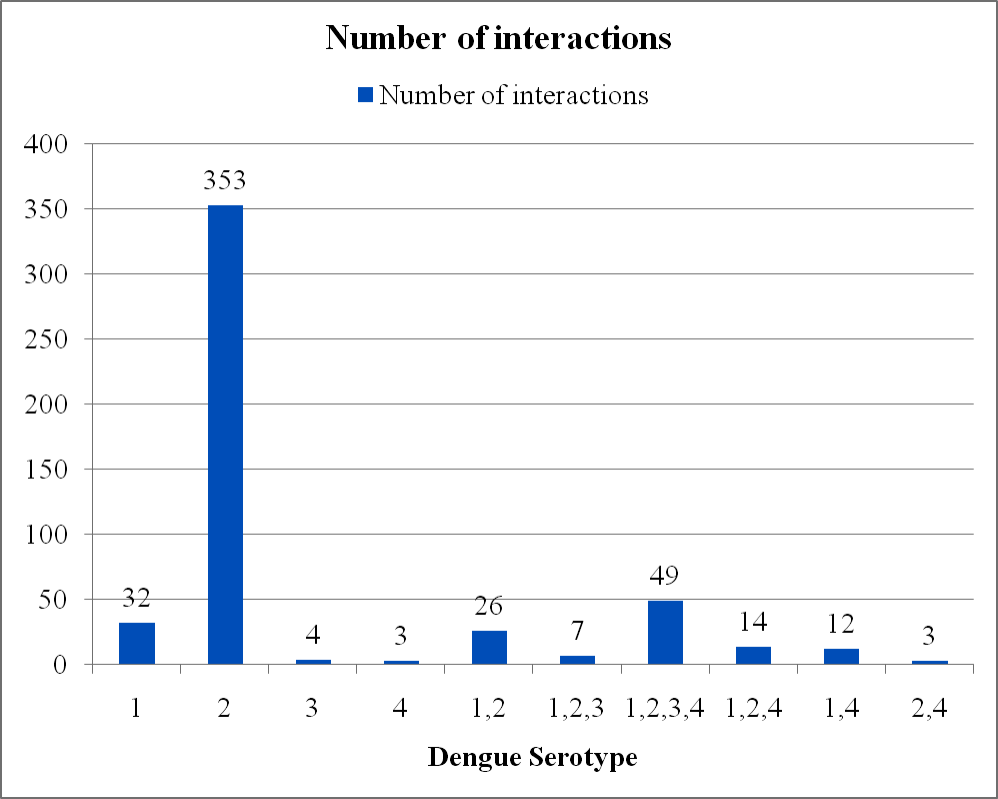
Number of dengue–human interactions with the corresponding proteins, with 1, 2, 3, 4, or a subset of serotypes. Dengue serotype 2 interacts with maximum number of human proteins (353).

**Fig 8:**
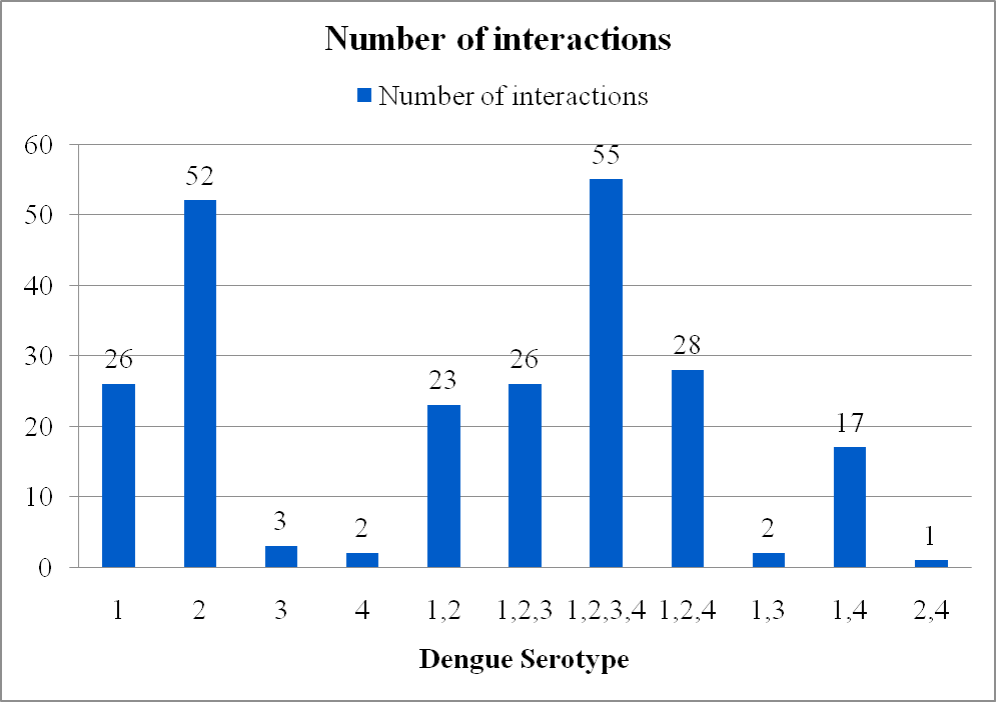
Number of dengue–mosquito interactions with the corresponding proteins, with 1, 2, 3, 4, or a subset of serotypes. Dengue serotypes 1–4 interact with maximum number of mosquito proteins (55).

The 4 serotypes of DENV are DENV-1, DENV-2, DENV-3, and DENV-4. Most of the dengue–host protein interactions are serotype specific. However, there is little information available about how each serotype differentially infects the host cells. According to Lindenbach et al. [25], around 65% of amino acid sequences are homologous among the 4 serotypes. If a dengue–host interaction involves multiple serotypes, then it may be said that the interaction is functionally relevant [15]. Several studies showed that after recovering from an infection caused by 1 dengue serotype, a person has immunity against only that particular serotype. Later on, that person can be infected with any of the remaining 3 dengue serotypes. Therefore, serotype-specific dengue–host PPI study is very important for understanding the vector pathogenesis. Most of the literature has focused on dengue serotype 2 for experimental analysis. Among the 535 dengue–human and 249 dengue–mosquito interactions, we identified serotypes of 503 and 235 interactions, respectively, from curated literature. For the rest of the interactions, we did not find any significant information from the associated literature reviews. A minority of the human proteins interacted with all 4 serotypes or a subset of the serotypes. We found that only 10% of dengue–human and 23% of dengue–mosquito interactions are serotype independent because host proteins interact with all dengue serotypes, and the rest of the interactions are serotype specific. A detailed list of the number of human and mosquito proteins that interacted with the corresponding proteins with 1, 2, 3, 4, or a subset of serotypes are given in Fig 7 and Fig 8, respectively. It is apparent from Fig 7 that dengue serotype 2 interacts with the maximum number of human proteins (353). More investigation is required to validate the rest of the serotype-specific interactions.

In this article, we have developed a database for PPI between dengue and its hosts curated from the literature and a variety of virus databases such as VirusMentha and VirHostNet. The advantages and disadvantages of this study are listed in Table 2.

**Table 2:** Advantages and disadvantages of this study.

Advantages	Disadvantages
1. This is the first database for collecting PPI between DENV and both its human and mosquito hosts.	1. In this study, we have considered only PPI between dengue and its host proteins; RNA–protein interactions are not considered here.
2. We have developed a web-based interface so that users can browse the database by choosing a single dengue or host protein.	2. All the dengue interactions are serotype specific, but some serotype information is missing from the database because we prepared the interaction database from the published literature. In some articles, the serotype information is not given.
3. Users can download the complete database from the website, and there is a feedback menu where users can give their suggestions.	3. The database is manually prepared from the published literature, so there may be some interactions not included. We will update the database periodically.

Abbreviations: DENV, dengue virus; PPI, protein–protein interactions.

**Fig 9:**
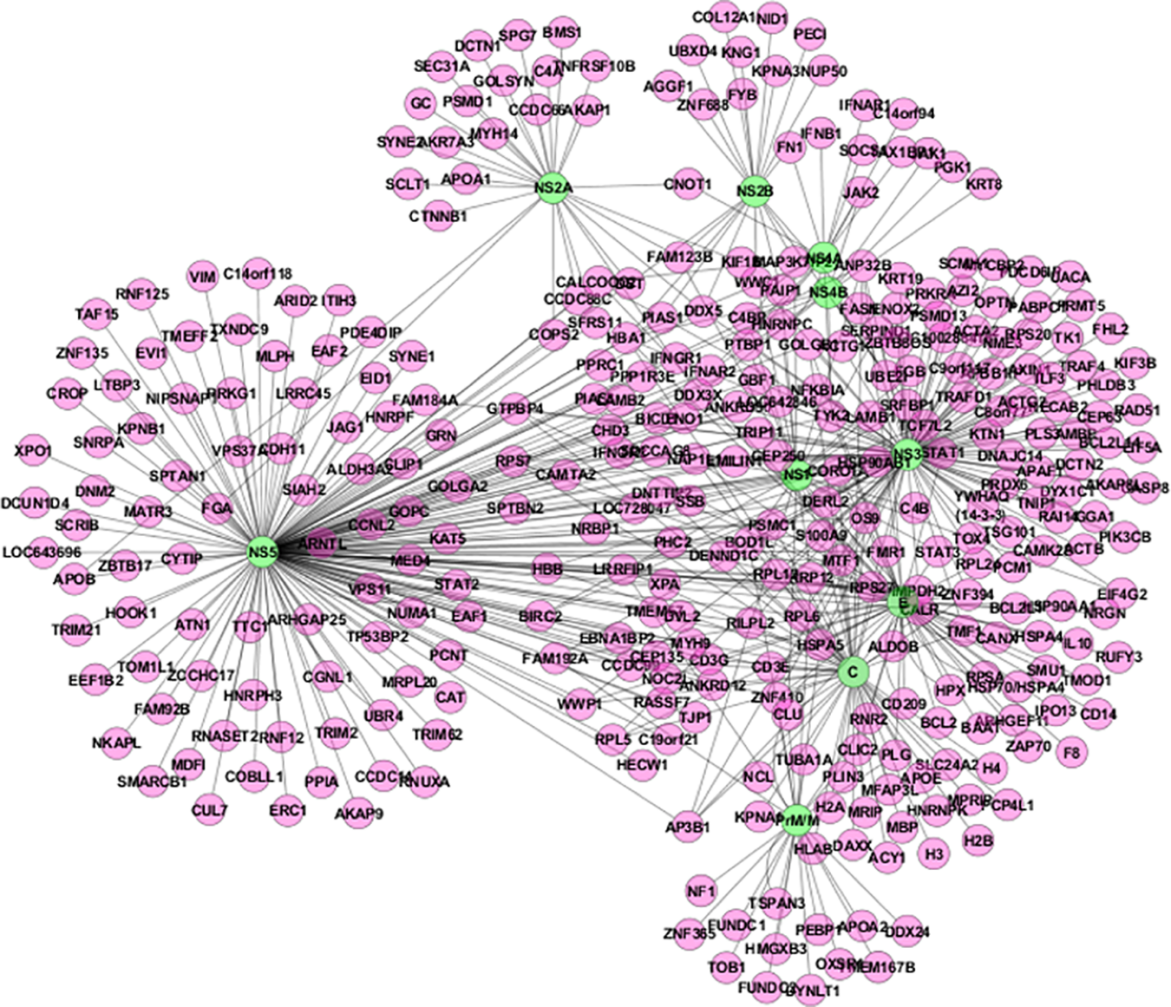
Dengue–human PPI network. A complete set of dengue–human protein interactions identified in this study is shown. Green ovals indicate dengue proteins, pink ovals indicate human proteins, and black lines indicate dengue–human protein interactions. PPI, protein–protein interactions.

**Fig 10:**
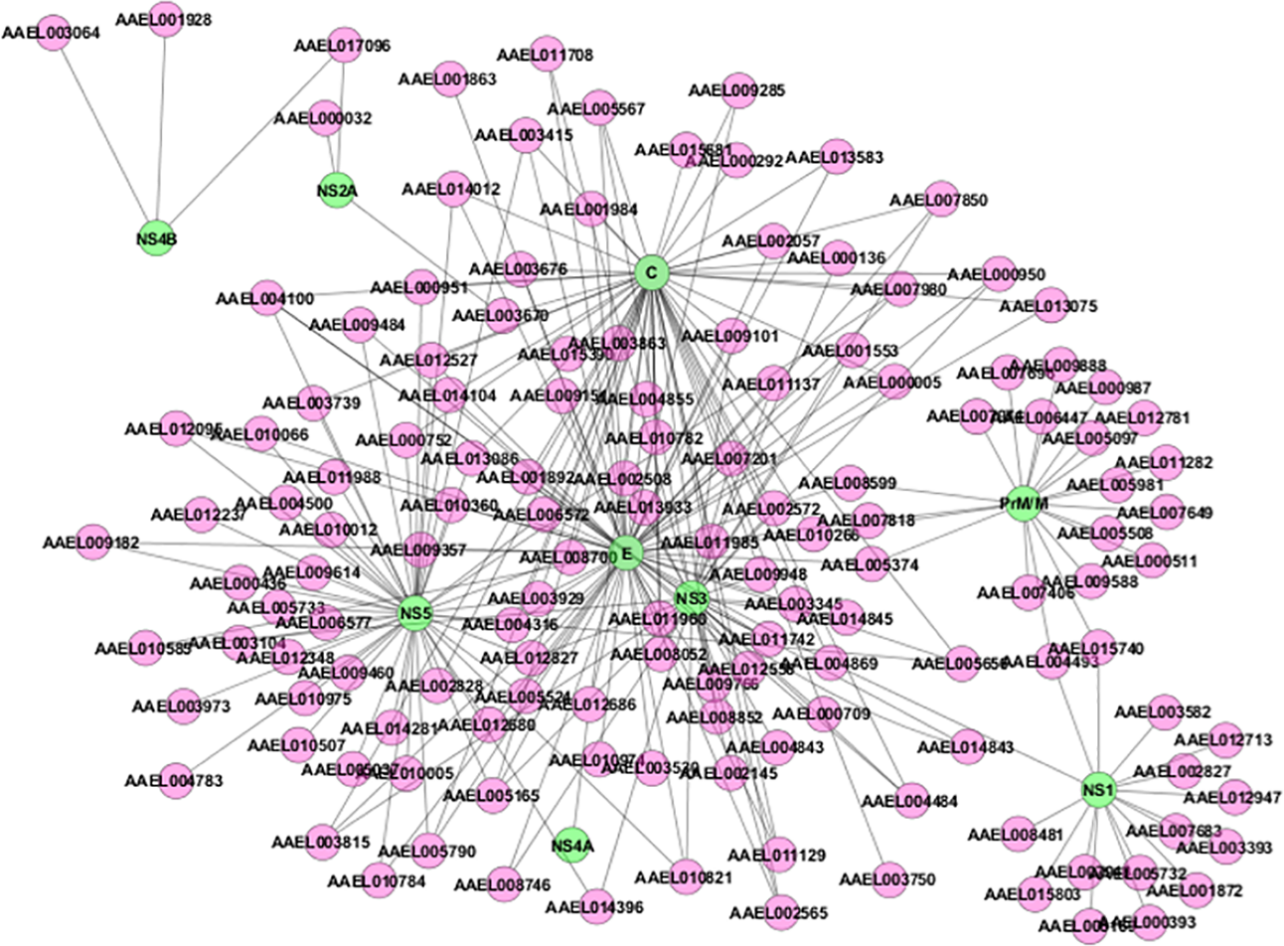
Dengue–mosquito PPI network. A complete set of dengue–mosquito protein interactions identified in this study isshown. Green ovals indicate dengue proteins, pink ovals indicate mosquito proteins, and black lines indicate dengue–mosquito protein interactions. PPI, protein–protein interactions.

A summary of PPI networks of dengue–human and dengue–mosquito identified in this study is shown in Fig 9 and Fig 10, respectively, using Cytoscape [23]. In the protein interaction graph, the nodes represent proteins, and the edges represent the existing PPI. The edges of the PPI networks are treated as undirected. The dengue–human interaction network includes 535 unique interactions between 10 dengue proteins and 335 different human proteins, and the dengue–mosquito interaction network includes 249 unique interactions between 10 dengue proteins and 140 different mosquito proteins. All interactions are currently supported by multiple forms of evidence, and those are mentioned in S1 File and S2 File.

## Conclusion

We have developed a dengue protein interaction database, DenvInt, a public repository, composed of both dengue–human and dengue–mosquito PPI networks. It captures virus–host interactions from the published literature, and each interaction is also embedded with the dengue serotype and PMID. NCBI gene IDs are appended with each human and mosquito protein and hyperlinked to NCBI so that researchers may get all information, e.g., the ID of other databases, aliases, etc., for each protein. In this current version of the database, we have limited it to only PPI because we felt the need for a dedicated interaction database for DENV and its hosts. In the future versions of the DenvInt, we would include RNA-binding proteins that interact with the DENV RNA and make it a more comprehensive database for finding host factors required for DENV. This database will be periodically updated every month with newly deposited interactions. We expect that DenvInt will become a valuable resource in the field of dengue–host PPI and will help researchers to take one step forward towards the development of antiviral therapies.

## Supporting information

S1 File Excel file containing dengue virus–human PPI with the sources used to build this database. Abbreviation: PPI, protein–protein interactions.
(XLSX)

S2 File Excel file containing dengue virus–mosquito PPI with the sources used to build this database. Abbreviation: PPI, protein–protein interactions.
(XLSX)

S3 File Excel file containing degree distribution of 335 unique human proteins in dengue virus–human PPI. Abbreviation: PPI, protein–protein interactions.
(XLSX)

S4 File Excel file containing degree distribution of 140 unique mosquito proteins in dengue virus–mosquito PPI. Abbreviation: PPI, protein–protein interactions.
(XLSX)
